# Supplementary material for: An assessment of the teacher completed ‘Early Years Foundation Stage Profile’ as a routine measure of child developmental health
Source: PLoS One. 2025 Mar 19;20(3):e0302771. doi: 10.1371/journal.pone.0302771 (PMC11957556; doi:10.1371/journal.pone.0302771)
Supplement: S1 File — (DOCX) [file pone.0302771.s001.docx]

**Supplementary File 1**

**Table 1:** Overview of the Early Learning Goals (ELGs) in the old and new versions of the Early Years Foundation Stage Profile (EYFSP)

| **Area of Learning** | **“Old” EYFSP ELGs (analysed version, delivered 2012-2021)**  (See Section 6 of 2021 EYFSP handbook for further detail) | **“New” EYFSP ELGs (new version, delivered since 2021)**  (See Section 3.4 of 2021 EYFSP early adopter handbook for further detail) |
| --- | --- | --- |
| Communication and language development | 1. Listening and attention  2. Understanding  3. Speaking | 1. Listening, attention and understanding  2. Speaking |
| Physical development | 4. Moving and handling  5. Health and self-care | 3. Gross motor skills  4. Fine motor skills |
| Personal, social and emotional development | 6. Self-confidence and self-awareness  7. Managing feeling and behaviour  8. Making relationships | 5. Self-regulation  6. Managing self  7. Building relationships |
| Literacy | 9. Reading  10. Writing | 8. Comprehension  9. Word reading  10. Writing |
| Mathematics | 11. Numbers  12. Shape, space and measures | 11. Number  12. Numerical patterns |
| Understanding the world | 13. People and communities  14. The world  15. Technology | 13. Past and present  14. People, culture and communities  15. The natural world |
| Expressive arts and design | 16. Exploring and using media and materials  17. Being imaginative | 16. Creating with materials  17. Being imaginative and expressive |

*Note: In the version of the EYFSP that we analyse in this study (second version, delivered 2012-2021), the EYFSP is scored according to whether a child meets each ELG as “Emerging”, “Expected” or “Exceeding”. A revised version of the EYFSP has been available since 2021, with minor changes to wording on the ELGs, and children are scored as only “Emerging” or “Expected”.*
