# Supplementary material for: An assessment of the teacher completed ‘Early Years Foundation Stage Profile’ as a routine measure of child developmental health
Source: PLoS One. 2025 Mar 19;20(3):e0302771. doi: 10.1371/journal.pone.0302771 (PMC11957556; doi:10.1371/journal.pone.0302771)
Supplement: S2 File — (DOCX) [file pone.0302771.s002.docx]

# **Technical Appendix File 1: Predictive Validity Analysis**

## Attachment A: Description of latent socioeconomic groups

| **Class** | **Description** |
| --- | --- |
| Least socioeconomically deprived and most educated” | Women currently and previously employed Father non-manual employment Women and fathers highly educated Up to date with bills Mortgage Not subjectively poor Not receiving means tested benefits Not materially deprived |
| “Employed, not materially deprived” | Women currently employed Father manual and non-manual employment Women and father medium levels of education Up to date with bills Mortgage Not subjectively poor Not receiving means tested benefits Not materially deprived |
| “Employed, no access to money” | Women currently and previously employed Father manual and non-manual employment Women and father’s medium levels of education Moderate behind with bills Mortgage and private renting Moderate subjective poverty Moderate receipt of means tested benefits Materially deprived in particular can’t afford holidays, money to replace goods and savings |
| “Benefits and not materially deprived” | Women low current employment Father manual employment and self-employed Women and father’s low levels of education, father’s education high don’t know response Up to date with bills Owns house outright Not subjectively poor High receipt of means tested benefits Not materially deprived |
| “Most economically deprived” | Women low current employment Father manual employment and unemployed Women and father’s low levels of education, father’s education high don’t know response Behind with bills Private renting and social housing Subjectively poor Highest receipt of means tested benefits Materially deprived |

##
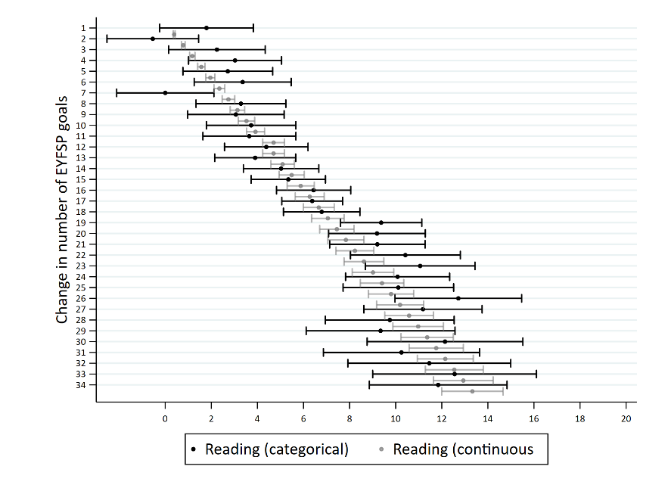

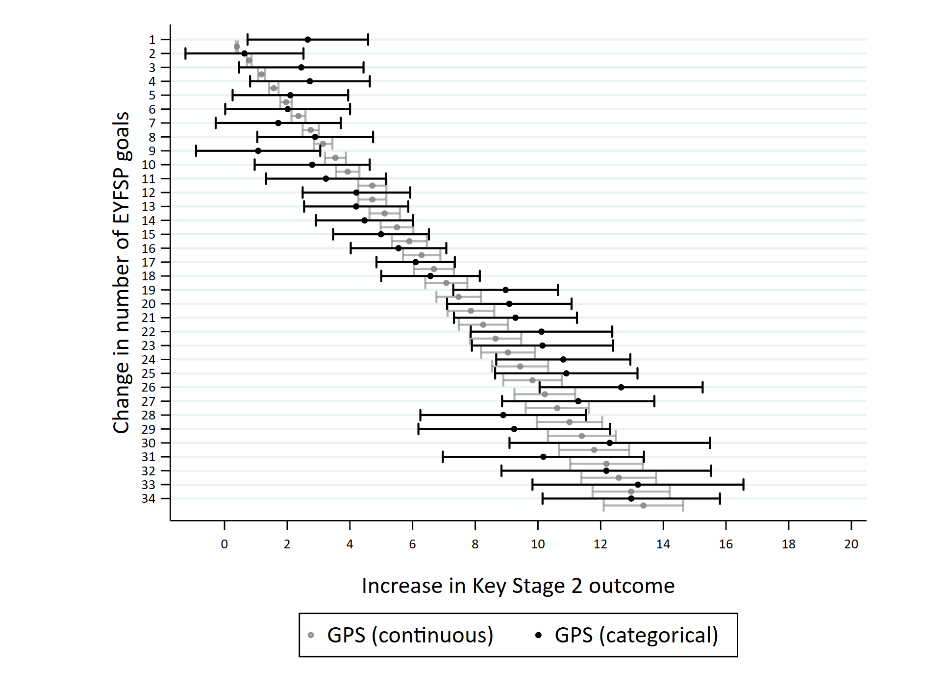

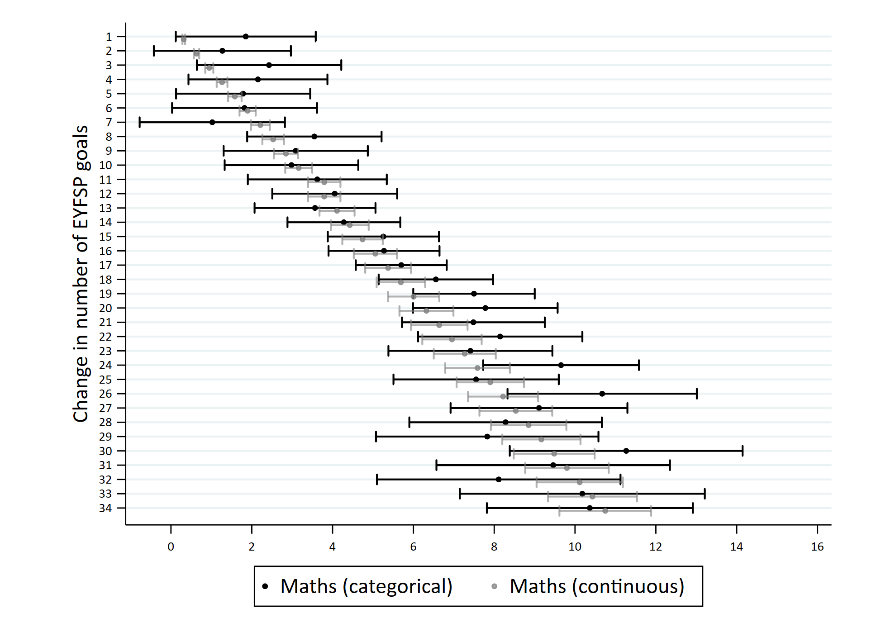
Attachment B: Categorical predictor (EYFSP)

Figures 5a-5b. Estimates when EYFSP is modelled as a (1) continuous (in gray) and (2) categorical predictor on Key Stage 2 outcomes

Model fit values for the continuous predictor were: Maths (AIC: 16997.45, BIC: 17080.12), Reading (AIC: 17901.71, BIC: 17984.38), and GPS (AIC: 17481.88 BIC: 17564.55).

Model fit values for the categorical predictor were as follows: Maths (AIC: 17025.04, BIC: 17302.58), Reading (AIC: 17910.96, BIC: 18188.5), and GPS (AIC: 17475.78, BIC: 17753.32).


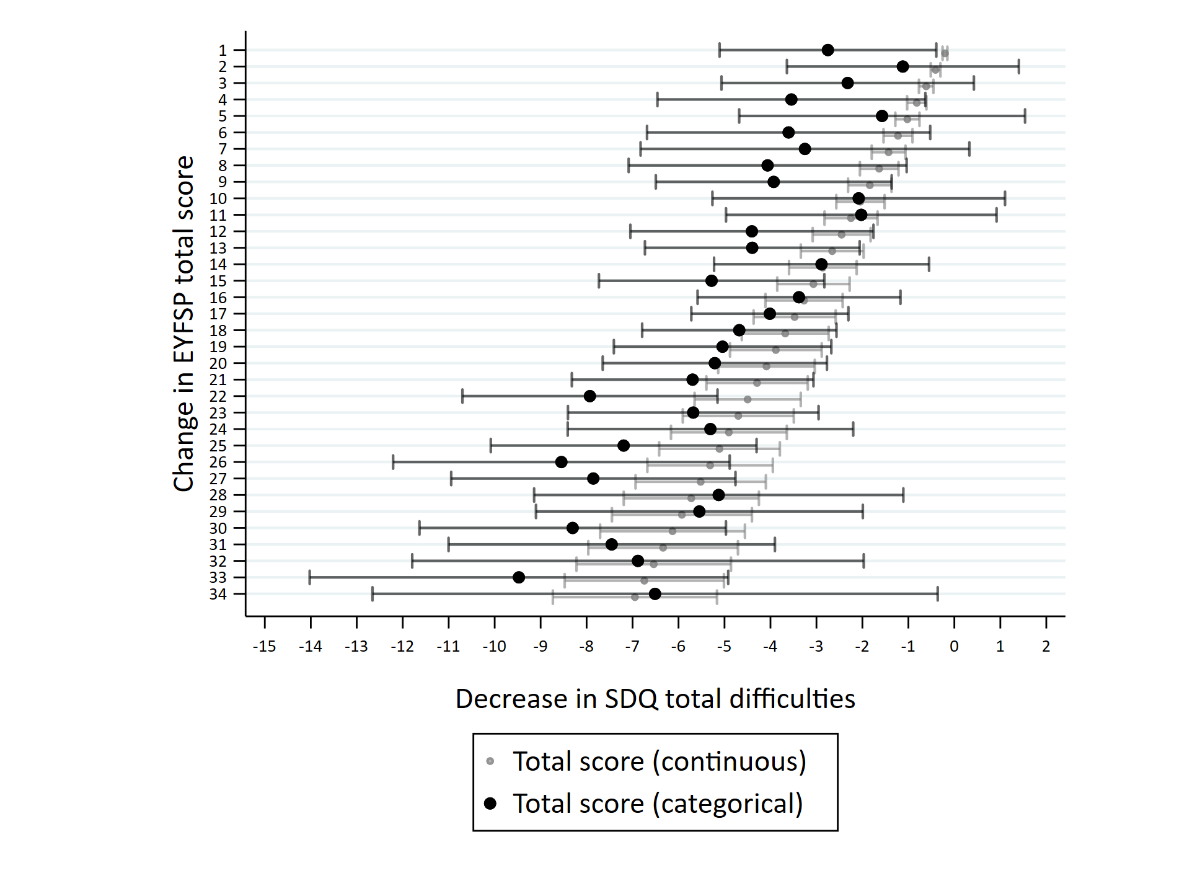


Figure 6a. Estimates when EYFSP total score is estimated as a (1) continuous (in gray) and (2) categorical predictor on SDQ total difficulties

Model fit values for the continuous predictor were AIC: 6124.798, BIC: 6193.281.

Model fit values for the categorical predictor were AIC: 6161.083, BIC: 6390.99.

Figure 6b. Estimates when EYFSP-CS score is estimated as a (1) continuous (in gray) and (2) categorical predictor on SDQ total difficulties


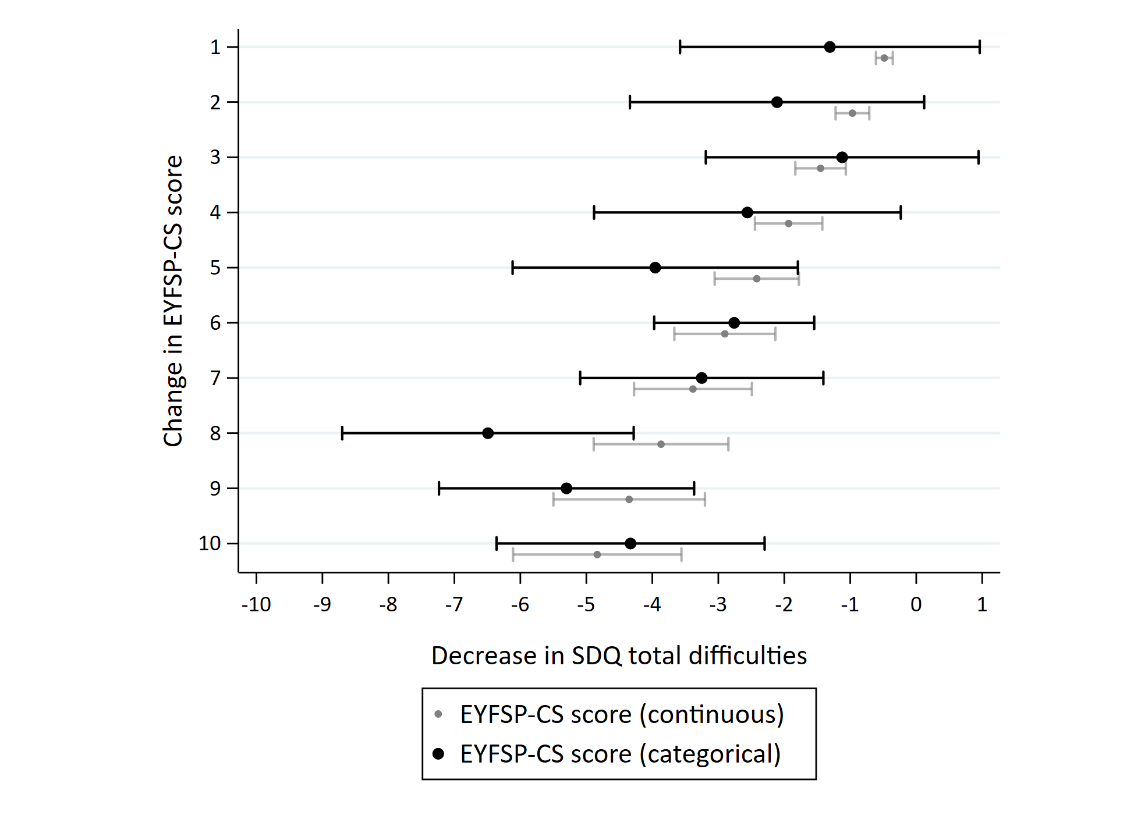


Model fit values for the continuous predictor were AIC: 6127.794, BIC: 6196.277.

Model fit values for the categorical predictor were AIC: 6135.962, BIC: 6258.252.

## Attachment C: Regression results for Key Stage 2

Table 7. Regression results for Key Stage 2 outcomes: (1) Maths, (2) Reading, and (3) Grammar, Punctuation and Spelling

| **Variable** | **Maths** | | **Reading** | | **Grammar, Punctuation and Spelling** | |
| --- | --- | --- | --- | --- | --- | --- |
|  | **B [95% CI]** | ***p*** | **B** | **p** | **B** | **p** |
| **EYFSP total score** | 0.356 [.322 to .390] | <.001 | 0.424 [0.384 to 0.464] | <.001 | 0.427 [0.390 to 0.464] | <.001 |
| **Ethnicity** |  |  |  |  |  |  |
| White British (reference group) | . | . |  |  |  |  |
| Pakistani | 0.805 [.017 to 1.593] | 0.045 | 0.349 [-0.573 to 1.271] | 0.458 | 1.786 [0.915 to 2.656] | <.001 |
| Other | 0.965 [0.238 to 1.692] | 0.009 | 0.878 [0.022 to 1.734] | 0.044 | 1.856 [1.057 to 2.656] | <.001 |
| **Parent place of birth** |  |  |  |  |  |  |
| Born in UK (reference group) | . | . |  |  |  |  |
| Born outside UK | 0.529 [-0.065 to 1.123] | 0.081 | 0.467 [-0.225 to 1.159] | 0.186 | 0.931 [0.275 to 1.588] | 0.005 |
| **SEP** |  |  |  |  |  |  |
| Most deprived (reference group) | . | . |  |  |  |  |
| Benefits but coping | 0.207 [-0.494 to 0.908] | 0.563 | -0.113 [-0.936 to 0.710] | 0.788 | 0.329 [-0.454 to 1.111] | 0.410 |
| Employed, no money | 1.611 [0.737 to 2.486] | <.001 | 1.115 [0.086 to 2.145] | 0.034 | 1.941 [0.999 to 2.882] | <.001 |
| Employed, not mat dep | 1.020 [0.236 to 1.804] [ | 0.011 | 0.375 [-0.617 to 1.367] | 0.459 | 1.227 [0.301 to 2.152] | 0.009 |
| Least dep, most educated | 2.884 [2.030 to 3.738] | <.001 | 2.438 [1.411 to 3.466] | 0.000 | 3.016 [2.047 to 3.985] | <.001 |
| **EAL** |  |  |  |  |  |  |
| No (reference group) | . | . |  |  |  |  |
| Yes | 0.334 [-0.324 to 0.993] | 0.320 | 0.030 [-0.746 to 0.806] | 0.940 | 0.301 [-0.422 to 1.023] | 0.415 |
| **SEN** |  |  |  |  |  |  |
| No (reference group) | . | . |  |  |  |  |
| Yes | -5.059 [-5.646 to -4.471] | <.001 | -5.276 [-5.970 to -4.582] | <.001 | -5.960 [-6.601 to -5.318] | <.001 |
| **Age in months** | -0.119 [-0.182 to -0.055] | <.001 | -0.109 [-0.184 to -0.034] | 0.004 | -0.124 [-0.193 to -0.055] | <.001 |
| **School (level)** | **2.151 [1.82 to 2.544]** |  | **2.353 [1.980 to 2.799]** |  | **2.771 [2.364 to 2.248]** |  |

### Code used in Stata-17 to produce above results:

*************************************

*********1. MATH ANALYSIS********

*************************************

*Obtain R2 values

mibeta maths_scaled eyfsptotal i.ethnicity3 i.pims i.sep i.eal i.sen agemonths_ks2 school

*Regression

mi estimate, saving(miestfile1) esample(esample) post: mixed maths_scaled eyfsptotal i.ethnicity3 i.pims i.sep i.eal i.sen agemonths_ks2 || school :

*************************************

*********1. READING ANALYSIS*********

*************************************

*Obtain R2 values

mibeta reading_scaled eyfsptotal i.ethnicity3 i.pims i.sep i.eal i.sen agemonths_ks2 school

*Regression

mi estimate, saving(miestfile2) post: mixed reading_scaled eyfsptotal i.ethnicity3 i.pims i.sep i.eal i.sen agemonths_ks2 || school :

*************************************

********* 1. GPS ANALYSIS *********

*************************************

*Obtain R2 values

mibeta gps_scaled eyfsptotal i.ethnicity3 i.pims i.sep i.eal i.sen agemonths_ks2 school

*Regression

mi estimate, saving(miestfile3) post: mixed gps_scaled eyfsptotal i.ethnicity3 i.pims i.sep i.eal i.sen agemonths_ks2 || school :

est store

## Attachment D: Regression results for SDQ

Table 8. Regression results for association between (1) EYFSP total score and SDQ and (2) EYFSP-CS score and SDQ

| **Variable** | **(1) SDQ (n = 984)** | | **(2) SDQ emotional ELGs (n = 984)** | |
| --- | --- | --- | --- | --- |
|  | **B [95% CI]** | ***p*** | **B [95% CI]** | ***p*** |
| **EYFSP total score** | -0.204 [-0.257 to -0.152] | <.001 | -0.483 [-0.611 to -0.365] | <.001 |
| **Ethnicity** |  |  |  |  |
| White British (reference group) | . | . |  |  |
| Pakistani | 0.300 [-0.992 to 1.593] | 0.649 | 0.341 [-0.951 to 1.633] | 0.605 |
| Other | 0.313 [-0.910 to 1.536] | 0.616 | 0.374 [-0.850 to 1.598] | 0.549 |
| **Parent place of birth** |  |  |  |  |
| Born in UK (reference group) | . | . |  |  |
| Born outside UK | -0.241 [-1.091 to 0.608] | 0.578 | -0.289 [-1.139 to 0.560] | 0.504 |
| **SEP** |  |  |  |  |
| Most deprived (reference group) | . | . |  |  |
| Benefits but coping | -0.926 [-2.051 to 0.198] | 0.106 | -0.891 [-2.018 to 0.236] | 0.121 |
| Employed, no money | -1.108 [-2.399 to 0.183] | 0.093 | -1.189 [-2.485 to 0.108] | 0.072 |
| Employed, not mat dep | -1.425 [-2.718 to -0.132] | 0.031 | -1.456 [-2.751 to -0.161] | 0.028 |
| Least dep, most educated | -1.992 [-3.304 to -0.681] | 0.003 | -2.095 [-3.404 to -0.785] | 0.002 |
| **EAL** |  |  |  |  |
| No (reference group) | . | . |  |  |
| Yes | -1.564 [-2.549 to -0.579] | 0.002 | -1.586 [-2.572 to -0.600] | 0.002 |
| **SEN** |  |  |  |  |
| No (reference group) | . | . |  |  |
| Yes | 4.566 [3.584 to 5.548] | <.001 | 4.730 [3.760 to 5.700] | <.001 |
| **Age in years** | 0.696 [0.068 to 1.324] | 0.030 | 0.646 [0.018 to 1.275] | 0.044 |
| **School (level)** | **1.436 [0.931 to 2.215]** |  | **1.42 [0.916 to 2.202]** |  |

### Code used in Stata-17 to produce above results:

*MICE regression: EYFSP as continuous*

**************************************

*Obtain R2

mibeta psy_sdq eyfsptotal i.ethnicity3 i.pims i.sep i.eal ageyears i.sen

*Regression

mi estimate, saving(miestfile11) esample(esample) post: mixed psy_sdq eyfsptotal i.ethnicity3 i.pims i.sep i.eal ageyears i.sen || psyschoolid :

est store S
