# Supplementary material for: An assessment of the teacher completed ‘Early Years Foundation Stage Profile’ as a routine measure of child developmental health
Source: PLoS One. 2025 Mar 19;20(3):e0302771. doi: 10.1371/journal.pone.0302771 (PMC11957556; doi:10.1371/journal.pone.0302771)
Supplement: Attachment — Submitted filename: Response to reviewers V1.0.docx [file pone.0302771.s003.docx]

**We thank all three reviewers for their reviews of the manuscript. We provide our response to each of the reviewers points below, with our responses written in blue. Where we refer to page numbers where we have made changes in the manuscript, the numbers relate to the manuscript with tracked changes.**

# Reviewer #1:

**Thank you for the opportunity to review the manuscript, “An assessment of the teacher completed ‘Early Years Foundation Stage Profile’ as a routine measure of child developmental health”, submitted to PLOS One.**

**The authors report a secondary data analysis, which attempts to establish the internal consistency and structural validity of the Early Years Foundation Stage Profile (EYFS-P) in one ethnically diverse region in England (i.e., Bradford).**

**In principle, this study has the potential to inform research using this specific administrative data. However, several substantial issues within the current manuscript prevent its publication in its current form and underscore my ‘reject’ recommendation. These issues are outlined in detail below. Overall, I hope this feedback is helpful for future revisions of this work.**

Response: We thank the reviewer for the thorough and rigorous review. We have made several changes to the manuscript based on their suggestions and feel it has strengthened it substantially.

**‘Developmental health’ definition and implications for structural validity evaluation**

**Throughout the manuscript, the authors refer to the EYFS-P as a measure of ‘developmental health’ which they define as “a broad concept that combines a holistic understanding of physical, mental, social and emotional wellbeing”.**

**However, this definition is not well-aligned with the intended purpose of the EYFS-P, which also incorporates “specific areas of learning” (i.e., early academic skills). For example, as highlighted in the DfE (2024) guidance, the EYFS-P is designed to assess the “prime areas of learning (which are: communication and language; personal, social and emotional development; and physical development) and the specific areas of mathematics and literacy” (as well as Understanding the world and Expressive arts and design) (see page 6 https://assets.publishing.service.gov.uk/media/65253bc12548ca000dddf050/EYFSP_2024_handbook.pdf). Moreover, the early learning goals (ELGs) within the EYFS-P are reflective of the early years foundation stage curriculum (see Development Matters documentation) and thus may be better thought of as a curriculum-based measure.**

**As a consequence of this misalignment, the authors have misattributed the constructs measured within the EYFS-P. This has important implications for their structural validity evaluation. Specifically, they have used a confirmatory factor analysis approach with the a-priori assumption that the EYFS-P loads onto one factor (‘developmental health’).**

**However, given the design and intended purpose of the EYFS-P, there may be alternative factor structures that should also be considered, for example, a 2-factor structure (prime areas of learning; specific areas of learning) or a 7-factor structure (Communication and language; Personal, social and emotional development; Physical development; Literacy; Mathematics; Understanding the world; Expressive arts and design). As such, an exploratory factor analysis approach would be more appropriate in the current study.**

Response: We appreciate the Reviewer’s point that the way in which we had defined ‘developmental health’ did not align with the intended purpose of the EYFSP, as it appeared to ignore some aspects of the curriculum that the EYFSP was based on. We revisited the reference for the term ‘developmental health’ (Keating et al., 1999) and found that they do include measures of mathematics and literacy in their definition. We have therefore amended the first sentence in our manuscript to ensure that the definition covers not only physical, social, and emotional wellbeing, but also “core educational abilities such as mathematics and literacy”. We feel that this clarification now better aligns with the original purpose of the EYFSP.

With regards to the issue around the confirmatory factor analysis (CFA) with all EYFSP items loading onto one factor, this was carried out as it is an essential analysis to accompany Item Response modelling. Item response models assume that the latent trait variable is reflected by a unidimensional continuum, thus this must be tested using CFA (explained in detail on p.14-15 of the manuscript). We do agree with the reviewer that an exploratory factor analysis of the EYFSP items would be interesting, to explore if the items do appear to relate to the areas they are intended to measure. However, since the intended purpose of our study was to validate the **total score** of the EYFSP (ie. not the EYFSP as a whole) – an EFA would not answer the objectives of our study.

**Application of the current findings to current educational practice**

**Although the authors used the 2008-2012 version of the EYFS-P as the basis for their study, they make the following conclusions: “We expect that the results from this study will generalise to the revised version of the EYFSP, particularly the findings regarding internal consistency and predictive validity” and “This study supports future use of the EYFSP total score over the EYFSP GLD score for research and educational purposes”.**

**These conclusions are not supported and are problematic because the 2008-2012 version of the EYFS-P is significantly different from current educational practice. For example, the most recent statutory EYFS-P introduced in Sept 2021 includes several substantial changes to the ELGs. These changes include the removal of the Shape, Space, and Measure ELG, for the introduction of the Numerical Patterns ELG, as well as distinct changes to the content of a range of the ELGs across the EYFS-P. These changes will have important implications for the psychometric properties that the authors have examined and thus limit the generalisability of the current findings.**

**Furthermore, it is of concern that the authors do not acknowledge anywhere in their manuscript that the EYFS-P was revised again in 2021 (e.g., on page 4).**

**The authors should, therefore, clarify that their results are only applicable to research studies that are planning to use the 2008-2012 EYFS-P total score (page 27). Any further application (without further investigation) would be inappropriate.**

Response: Thank you to the reviewer for raising concerns around the version of the EYFSP examined. We agree this is important, as the EYFSP has changed several times over the years, and agree it is crucial for the reader to understand which version is used.

We used the version of the EYFSP delivered between 2012-2021, not the version delivered between 2008-2012. We apologise for the confusion and believe this may be because we stated that we used the ‘original version’ of the EYFSP (p.6) – we have now updated this to say we investigate the ‘second version of the EYFSP’ (p.6). Based on the reviewer’s recommendation, we have also included a file (Technical Appendix File 1) which shows the second version of the EYFSP (the one we analysed), and the revised version (the one used in practice now). We have also made our statement clearer that the EYFSP was revised in 2021. We have also stated in the limitations of our discussion that we only considered data from this version of the EYFSP, and state that future research should test if the findings generalise to the newer version (p.34). We hope this alleviates the reviewers concerns around our conclusions.

**Fine-grained analytical approach**

**Although the authors helpfully adopt a more fine-grained approach for considering the relationships between EYFS-P Communication and Socioemotional goals and SDQ difficulties (page 25), there presents a missed opportunity for adopting a similar approach for literacy-based ELGs to later literacy outcomes, and maths-based ELGs to later maths outcomes. This is despite the authors’ argument on page 6, which states “this rationale can be generalised to all seven areas of the EYFS-P”.**

Response: We agree with the reviewer that it will be helpful to examine relationships between specific EYFSP goals and later outcomes. The Born in Bradford dataset facilitated an analysis of the longitudinal association between EYFSP goals and SDQ scores, and this is particularly useful as SDQ is a widely validated measure of children’s socioemotional abilities. However, data relating to all other areas of the EYFSP were not available in the Born in Bradford sample. Our discussion does highlight that ‘more research is needed to explore whether each specific goal area is associated with other measurements’, and we have now added in an explanation of why we selected the SDQ as the test case (p.35-36).

**Psychometric terminology**

**Pages 6-7- The authors write “information about the construct validity (i.e. the extent to which a test measures what it is intended to measure)… or instance, do the ‘personal, social and emotional development’ areas have significant predictive associations with a validated measure of children’s social and emotional development?” This is incorrect, as it is an example of (predictive) criterion validity, not construct validity. I recommend that the authors familiarise themselves with resources, such as the AERA, APA, NCME Standards for Educational and Psychological Testing, which will help ensure that the terminology they use is accurate.**

Response: Thank you for highlighting this mistake in our language. We agree with the Reviewer that this paragraph is describing predictive validity, and have updated the language to clarify this (p.8)

**Page 8- The authors write “this [internal consistency] is an essential first step prior to investigating the structural validity of the EYFS-P” – this is not strictly true. Rather the COSMIN taxonomy and the AERA, APA, NCME Standards for Educational and Psychological Testing resources (mentioned above) suggest that structural validity comes first (i.e., how many factors are there?), followed by internal consistency (i.e., are the items interrelated?). Furthermore, the interrelations between items should be considered at both the full-item level (i.e., all EYFS-P items) and (if applicable) at the specific factor level (e.g., if the structural validity evaluation identified 2-factors, then the internal consistency also needs to be reported for these 2 factors). As such, I recommend that the reporting of the results in the current study be re-structured (and re-analysed per the suggestions mentioned).**

Response: Thank you for raising this important point. We have now rearranged our analysis so that we first present the structural validity, followed by internal consistency.

We do appreciate the reviewers point around structural validity evaluation (e.g. potentially identifying >1 factor), however, in this case the EYFSP ‘total score’ measure is already being used – hence the structural validity section of our study aims to validate the total score only. We have explained this briefly in the rationale.

**Other specific issues within the manuscript**

**Page 3- The authors write “The embedding of standardised measurement of this into educational systems varies greatly across countries”, but then go on to discuss one example (Canada) – are there other examples that could be included here?**

We have now explained internationally used measures of child developmental health (the Early Child Developmental Index, and Early Development Instrument). We have also contextualised how these are embedded into educational systems – with the EDI into Canda and Australia, and the Teaching Standard Gold (TS GOLD) in the US (p.3-4).

**Page 3- The authors write “Due to the educational pressures that standardised exam settings can bring, assessments completed by children’s teachers can instead offer a valuable insight” – this is somewhat true, but requires further clarification- e.g., Educational pressures are also not the only reason why teacher-based assessments may be considered optimal with very young children. Likewise, teacher-based assessments can also be ‘high-stakes’, such as those completed in Key Stage 1.**

Response: Here we mean to refer to the stress that is caused to children by exam settings, and we have now clarified this. (p.5)

**Page 3- The authors write “The EDI… has generally demonstrated adequate psychometric properties in terms of internal consistency (15), and predictive validity”. It would be helpful to report exact figures here, as the term ‘adequate’ is often inconsistently and/or misused across psychometric studies. The COSMIN taxonomy and the AERA, APA, NCME Standards for Educational and Psychological Testing, will provide helpful starting points for the authors in establishing the consistent use of the term ‘adequate psychometric properties’.**

Response: We have added in the internal consistency, model fit, and predictive validity values for the measurement instruments that we describe (p.3-4).

**Page 4- The authors write “69 ‘early learning goals’ (ELGs)”- this is not strictly true, rather the DfE refer to the structure of the 2008-2012 version of the EYFS-P as “13 assessment scales, with 9 points within each scale (‘scale point’). The 13 assessment scales are grouped into six areas of learning” (e.g., https://assets.publishing.service.gov.uk/media/5a7b875c40f0b62826a0429c/sfr28-2010.pdf and** [**https://dera.ioe.ac.uk/id/eprint/8221/13/Early_FS_Handbook_v11_WO_LR_Redacted.pdf**](https://dera.ioe.ac.uk/id/eprint/8221/13/Early_FS_Handbook_v11_WO_LR_Redacted.pdf)**).**

Response: We appreciate the reviewer’s comments on this point, and we have revisited the literature on the reforms to the original EYFSP. Our reading of the Tickell Review (reference #17) and the government’s publications on the changes to the EYFSP (see here: <https://www.gov.uk/government/news/new-early-years-framework-published>) confirms our understanding that the original 69 early learning goals were consolidated into the 17 used in the second version of the EYFSP.

**Page 4- The authors write “the original version…has been used nationally and routinely for nine years” – this is also not strictly true, as the 2008-2012 version of the EYFS-P (the focus of the current study) was used for 4 years. The 2012-2021 revised version for a further 9 years, with the latest iteration made statutory in Sept 2021.**

Response: This relates to the earlier point regarding which version of the EYFSP was used, we have added in clarification that we are looking at the second version of the EYFSP, not the original version.

**Page 6- The authors highlight research evaluating the measurement properties of the EYFS-P, but do not include other relevant literature, e.g., Snowling et al. 2011 https://files.eric.ed.gov/fulltext/ED526910.pdf**

Response: We have now included reference to this study (p.6).

**Page 6- The authors highlight “there are seven individual learning areas within the EYFSP” – (this refers to the 2012-2021 version), but on page 4 the authors state “the present study investigates the original version” (which I assume to mean the 2008-2012 version), which would include 13 assessment scales (see above point)- further clarity is required here.**

Response: This relates to an earlier point regarding which version of the EYFSP was used, we have added in clarification that we are looking at the second version of the EYFSP, not the original version.

**It may also be helpful to create a table (perhaps expanding on table 1) that outlines each of the three versions of the EYFS-P, including which learning areas are measured, how they are measured (i.e., meets, exceeds, etc) and when they were implemented (i.e., years).**

Response: We agree with the reviewer that a table is helpful for clarifying the areas of learning, thank you for the recommendation. We have included a table which outlines the second version of the EYFSP (the version that we use) and the revised version of the EYFSP (the version currently used in practice). We have not included the original version of the EYFSP, as this would contain lots of information (69 ELGs), and we do not feel that this version has implications for our findings (uploaded as Technical Appendix File 1).

**Page 7- The authors write “In understanding these strengths and weaknesses, a child could then be provided with support in a particular area”- this argument needs to be further developed, as the EYFS-P in its current scoring structure is still designed and used for this purpose by educational practitioners. How is what the authors are suggesting, any different (and superior) from current practice?**

Response: We have made amendments here to explain that teachers already do this, and that our findings would provide confidence to this practice.

**Page 15- The authors write “Analysed scores therefore ranged between 80 and 120”- It is not clear how academic attainment was measured - were maths, reading, and grammar considered separately, or combined? what score was used- raw or scaled? it isn't clear why 80 is the minimum score?**

Response: We have made minor amendments to clarify that scores were analysed separately. Scores are obtained from educational records, and these range between 80-120 (p.18). The website that we reference (ref #57) explains this further:

- GOV.UK. Understanding scaled scores at key stage 2 - GOV.UK [Internet]. 2019 [cited 2023 Feb 23]. Available from: https://www.gov.uk/guidance/understanding-scaled-scores-at-key-stage-2#what-is-a-scaled-score

**Pages 15-16- Further clarity is required about the SDQ measure- e.g., were children assessed every year between the ages 7-10? Or just once? What are the five scales? Is the prosocial scale included in the five scales?**

Response: We have now clarified that the SDQ was measured just once per child and included mention of the four scales. As already stated, the prosocial scale is not included in the total difficulties score (p.18).

**Page 16 – What is your evidence to justify the inclusion of these covariates? “thought to be confounders” is not evidence-based.**

Response: We have now stated in table which variables are thought to be confounders of the association between EYFSP and the outcomes and included at least one reference to a study which justifies its inclusion (p.20).

**Page 20- There is a relatively large proportion of missing data within the socioeconomic position variable (18%), which is comparable to the size of other response options. Although missing data methods are applied, it would be beneficial to conduct additional robustness checks with other socioeconomic-proxy variables, such as children’s eligibility for free school meals or pupil premium. These administrative data are likely to be available for a larger proportion of children in the linked sample that the authors are already using.**

Response: As noted by the reviewer, there is some non-negligible degree of missingness in the socioeconomic status (SES) variable. We have attempted to address potential selection bias due to these missing values by multiply imputing these under the assumption that they are missing at random conditional on the other variables included in the analysis model and a binary variable indicating eligibility for free school meals (FSM) which was fully observed. We have added a sentence to the ‘Missing data methods’ subsection to clarify that FSM was included as an auxiliary variable in the imputation model and apologise for omitting this important detail from the original submission. We also note that the SES variable was included in the substantive analysis model to mitigate against confounding (as opposed to it being an exposure variable of substantive practical interest) and that the proportion of missing cases was relatively small (albeit higher than the other variables). Hence any systematic non-random departures from the MAR assumption that underpins our analysis would need to be quite extreme to have a material impact on the key results and conclusions.

As noted above, we have access to complete data on eligibility for free school meals and used this data to impute the missing covariate data in our planned analyses. While we agree that in some samples it could be beneficial to conduct a sensitivity analysis using eligibility for free school meals as a proxy for SES, members of the Born in Bradford team have generally found that this data is not reliable for measuring socioeconomic position for families in Bradford (e.g. Yang et al., 2022). Many families, particularly ethnic minorities, who are eligible for FSM do not apply, and many families who experience food insecurity are not eligible for FSM. We therefore decided when planning the analyses that a sensitivity analysis using this indicator of SES is unlikely to add robustness to our findings, since the measure is not sensitive to socioeconomic position. However, given that we have data on eligibility for FSM, we have undertaken additional post-hoc analyses to investigate the impact of conditioning on this variable in place of the multiply imputed SES variable for the analyses of each of the academic attainment outcomes. The estimated coefficients for the EYFSP total score were similar to those obtained from the planned analyses for all three of the academic attainment outcomes. We have chosen not to add this to the manuscript as we feel the analysis is already very long, however we can provide these results to the reviewer on request.

- Yang, T.C., Power, M., Moss, R.H., Lockyer, B., Burton, W., Doherty, B. and Bryant, M., 2022. Are free school meals failing families? exploring the relationship between child food insecurity, child mental health and free school meal status during COVID-19: national cross-sectional surveys. *BMJ open*, *12*(6), p.e059047.

**Page 20- Were there missing data for other predictor and outcome variables (described on pages 15-16)? If so, how were they handled? What proportion of data were missing?**

Response: There were missing data in the predictor and outcome variables described, the values are provided in Table 4 (page 24) and Figure 1 (page 24). The method for handling missing data is described, which was multiple imputation using chained equations (page.21). Due to Reviewer 2’s comment, we have also now justified our use of multiple imputation for this study (p.21)

**Page 30- The authors helpfully highlight the ethnic diversity of their sample. However, the authors should expand their discussions to consider any potential SES/ethnic inequalities in teacher-based assessment (rather than child-direct measures).**

Response: Thank you for raising this important point. This is certainly of interest to us, and we feel this is very important for a future study on the EYFSP. We do feel that a discussion of this is beyond the scope of the current manuscript, and we did not test for any differences across ethnic groups so cannot comment on the inequalities in the EYFSP. However, due to the reviewer noting this, we have now stated this as a limitation (p.34), and included a sentence to suggest this for future research (p.36).

**Page 31- The conclusion “total score is an internally consistent measure with predictive validity” should be further clarified/toned down, as it is only for the very low and very high-performing children; the 'average' child is not adequately assessed. Although the authors later add “we caution against using it for measurement of children with very close to ‘average’ ability levels” – the first highlighted sentence is misleading for readers.**

Response: Despite the limitations of the EYFSP for measuring ‘average’ ability children, we believe that the conclusion that the total score is internally consistent and has predictive validity is accurate, due to the McDonald’s hierarchical omega value being 0.89, and due to the significant associations between EYFSP and later outcomes. However, we do a acknowledge that is important to emphasise the limitations of the scale in the manuscript. We have therefore restructured the conclusion to ensure that readers are aware of the limitations of the EYFSP for measuring children with close to average abilities first and foremost, before describing the score as internally consistent and having predictive validity. We have done so in both our conclusion (p.36), and the abstract (p.2).

**Minor points**

**Page 3 – “can therefore improve” would be better phrased as “can therefore help contribute to” – or something similar. Currently, the text implies a definitive causal relationship, which excludes the possibility of other factors contributing to later, positive child outcomes.**

Response: We have made this change (p.3).

**Page 3- “kindergarten teachers [in Canada]”- what age is this?**

Response: We have clarified that this is when children are aged 4-6 years old (p.3).

**Page 8- “future outcomes” – which ones?**

Response: It is stated which future outcomes we investigate at the end of the paragraph on page 10.

**Page 10- “an observational birth cohort” – additional text is required to clarify the data is from one region in England (Bradford).**

Response: We have made this change (p.12).

**Page 10- “for all women recruited” – however the authors also mention fathers in paragraph 2, page 10. Please clarify the text accordingly.**

Response: We have made a change to reflect that written consent was obtained for ‘all parents recruited’ (p.12).

**Page 15- “two key changes to this upon starting the analysis” – what was the rationale for these changes?**

We have now included the rationale for the changes to our pre-registered analysis (p.17).

**Page 15 (and throughout the manuscript)- inconsistent use of SEN/ SEND.**

Response: We have made this change to consistently refer to SEND throughout.

**Page 15- make it clear that the “Key Stage 2 assessment” is completed by children, and not a teacher-based assessment.**

Response: We have made this change.

**Page 15- “end of Year 6 at school”- what age is this?**

Response: We have clarified this is when children are aged 10-11 years old (p.18).

**Page 16- use APA guidelines when using numbers in text – i.e., five, instead of 5.**

Response: We have made this change throughout.

**Page 19- “Model fit assessed via AIC and BIC was marginally better with EYFSP as a continuous variable, and the continuous modelling provides a more parsimonious estimate, so this model was selected” – please clarify where these results are reported.**

Response: We have now added model fit values for all models to Technical Appendix File 2 (previously File 1).

**Page 23 (and throughout the manuscript)- the information reported on the OSF page should also be included in a Supplementary materials section in the published manuscript.**

Response: We have now uploaded the pre-registration of our study as a supplementary material. We cannot upload the HTML file of our results, as PLOS ONE does not accept this format of files. This file is only available on the OSF page.

**Page 24- “EYFSP total score was associated with a higher Key Stage 2 outcome” – This text needs to be clarified – e.g., “higher EYFS-P total scores were…”**

Response: We have made this change (p.28).

**Page 24- what does GPS stand for? This is the first time the acronym is used.**

Response: At the top of page 24, we define the GPS abbreviation by saying “*The mean scores and standard deviations for the Key Stage 2 outcomes were … Grammar/Punctuation/Spelling (GPS)*”.

**Page 25- “Note that a higher score on SDQ indicates more socioemotional difficulties” should be included in the text on page 16 (i.e., SDQ method section).**

Response: We have made this change (p.18).

**Page 29- “affected by the removal of one of the response categories” – be clear which one.**

Response: We have made this change (p.34).

# Reviewer #2:

**This manuscript examines the internal consistency and structural and predictive validity of the EYFSP measure using linear mixed effects models, a CFA, and an IRT. This is thorough and timely work, and the statistical tests in the manuscript are appropriate and well-described. The feedback that I have is intended to strengthen the manuscript for publication.**

Response: We thank the reviewer for the thorough and rigorous review, and the appreciation of the work. We have responded to each of their comments below and made several changes to the manuscript which has substantially strengthened it.

**1. In the predictive validity analysis on page 15, please provide a brief justification for the changes made to the pre-registered analysis.**

We appreciate the reviewer highlighting this to us. We have now included the rationale for the changes to our pre-registered analysis (p.17).

**2. Please provide a brief justification for the use of MICE over other missing data imputation methods (i.e., why a MAR method over a MCAR method?).**

Response: We thank the reviewer for highlighting this, and we have now made some amendments and additions to the ‘Missing data methods’ subsection to more explicitly state the dependence of our analyses/results on the validity of our assumptions about the missing covariate data (p.21-22). We also provide more detail on our decision below.

We chose to use multiple imputation to address missing data among the covariates of the analysis model since for the present case, the assumption that these missing data are missing at random (MAR) is a priori more plausible than the much more restrictive assumption that these data are missing completely at random (MCAR). We note that the assumption that the data are MCAR could potentially be refuted using the data, however failure to reject the hypothesis that the data are MCAR would not constitute proof that these data are MCAR. Even if the data truly are MCAR, the analysis using multiply imputed data is not materially compromised compared to an analysis of only the complete cases (assuming that the imputation model is not severely mis-specified).

We chose to use MICE specifically, because this method provides the flexibility to impute several variables of different types (in our case two binary variables and an ordered categorical variable) which feature non-monotone patterns of missingness, without needing to explicitly specify a joint model for the observed and missing data.

While the validity of our analysis rests on various assumptions relating to the appropriateness of the imputations and the substantive analysis model, we believe our analysis is less likely to result in biased estimates of regression coefficients and their standard errors compared with an analysis that either excludes the cases with missing covariate data, or imputes these data as if they were known to be MCAR (especially if just a single set of imputed values is used).

**3. I would like if the implications of the misfit of certain items, as demonstrated by the RMSEA scores and discussed on pages 22-23, were more directly addressed in the discussion. Why might these particular items have misfit issues or be particularly impacted by the issues addressed by the authors on pages 27-28?**

Response: Thank you for raising this. It has strengthened our discussion and highlighted an important point for future research. We have now added discussion of the worst fitting items p.32.

**4. The authors mention several times that caution should be taken when making inferences about 'average' ability children. I wondered if the authors believe that there is an alternative to the EYFSP total score worth exploring for these children, and what this might be if so.**

Response: Thank you for raising this important point. We believe replacing the EYFSP with another measure would bring a significant challenge and overhaul to the educational system and would suggest avoiding this until the measurement properties of the EYFSP have been further explored. We have now explained this on p.36.

**5. In their limitations, the authors mention that the BiB cohort may not be relevant across other kinds of populations. In what ways do the authors believe that this sample may yield different results as opposed to other samples?**

Response: Thank you for helping us to expand on this in our discussion. One of the reasons the findings regarding the EYFSP may be different in BiB is that BiB contains a much more ethnically diverse sample that the general population. We have now explained in the ‘implications and future directions’ section how the EYFSP may yield different results over different populations (ie. due to teacher-based assessment potentially effecting assessment of ethnic minority groups) (p.36).

**6. Along the same lines as the above, in the methods and materials section, the authors should provide more detail about the demographic makeup of the BiB cohort. They mention in the discussion that this is an ethnically diverse sample. Can specific ethnicity and/or SES demographics be included in the main manuscript beyond the inclusion of the two largest ethnicities represented?**

Response: Thank you for raising this important point. We agree that more detail about the demographic makeup for the BiB cohort is important for a reader. We have now:

- Mentioned the third and fourth most populous ethnic groups in the BiB cohort in the Setting section (p.12).
- Included description of the four most populous ethnic groups within the ‘Other’ group in Table 1 (p.24)

# Reviewer #3:

**The authors take on an extraordinary blind spot in the UK public policy with child development and education at the intersection. Millions of teachers complete millions of measures on millions of children, and never has a psychometric property been considered. The authors clearly list their intentions, and the rationale for the study is well articulated. The authors also thoroughly examine the precedent held where data were compressed into Good Level of Development. This reviewer agrees that the dichotomous representation bleaches the data of nuance.**

Response: Thank you to the reviewer for their understanding and appreciation of this work. We appreciate their helpful review of our study. We have responded to each of their comments below and made several changes to the manuscript which has substantially strengthened it.

**Little was written in the background as to how the EYFSP was developed, though the brief history of its revision is included. Some of the administration of the EYFSP methods are left to the discussion rather than the background. That the EYFSP is not standardized or moderated leaves the original intent (research) of the tool to collect dust. This reviewer is stunned that a gigantic, incalculable number of person hours is invested in the UK each year on a measure that was never "normed." This is not a criticism of the present manuscript, but rather a reason to accept this manuscript that critiques our well intentions even when they are not evidence-based. I was relieved to learn that there was indeed high internal consistency, but do wonder if the original authors of the measure (or panel, or work group - however it came to pass) developed the EYFSP with some degree of sound methodology.**

Response: We agree with the reviewer that it is stunning that the EYFSP was never psychometrically developed and/or normed, and we are pleased that they clearly understand the importance of this study. We have investigated the available documentation on the development of the EYFSP, and we have not found any in-depth description of how the items were chosen/developed, except for the very brief descriptions that we provide on pages 5-6.

**The authors present novel findings on the extended predictive properties of the total score, now reaching children 10-11. Some inconsistent arguments exist in that the total score is discussed as needing to increase by 8-10 in order to achieve 1/2SD on key stage 2 scores. That seems statistically true, but then again the total scores are discussed throughout the manuscript as lacking nuance. Also, the EYFSP is given once, correct? No change in scores at the individual level can be expected - it's a developmental health cross sectional measure. If this reviewer has missed a key point, and that they EYFSP is a longitudinally administered measure, please correct me.**

Response: It is correct that the EYFSP is only administered once, not longitudinally.

The total score lacks nuance for average ability children, which refers to children with scores between approximately 15 and 18. Therefore a difference of 8 points in the EYFSP total score would move children either out of or beyond this ‘average’ range. For instance, if an intervention were to increase expected EYFSP scores by 8 points, compared to a control group with an expected score of 15 – this difference lies beyond the ‘average’ ability children. We therefore believe that our conclusions concerning the predictive properties of the EYFSP total score are not affected by the limited information provided by this score when using it to discriminate between the abilities of approximately ‘average’ ability children.

Our argument around the increase in scores is that we hope this information will be helpful for assessing the effectiveness of interventions (when a treatment and control group exist and the EYFSP could be used as an outcome after the intervention). To clarify this, we have amended the language in our discussion to refer to a ‘*difference* in EYFSP goals’, rather than a ‘change’ – which we believe may be confused with the individual level (p.33-34).

**In limitations, I do not follow how the authors conclude that their findings will generalize to the revised version. The revised version seems to compress data even further, limiting total score nuance even further.**

Response: Thank you for helping us to consider this further. We believe that the findings regarding internal consistency and predictive validity are likely to generalise, as these should be less effected by removal of a response category – ie. the items should remain internally consistent, and predictive of future outcomes, even with less variation in data. However, we have now made the wording around this statement more tentative and included a more thorough explanation of it. We do agree that the structural validity is very likely to be affected by the removal of one of the response categories, and have now included a possible reason why (p.34).

**Implications and future directions: does this manuscript really support continued use of this measure, or should it be revised to support educational benchmarking and interventions? I felt a firmer stance was being taken in the discussion for the total score instead of the GLD score. This reviewer appreciates the authors consideration of how else the EYFSP should be examined. How should it change, though? With it being in its teen years after development, if the authors are calling for revisions to the method of examining it, can they also call for revisions to the tool itself?**

Response: Thank you for raising this. We believe our study should give teachers and researchers more confidence in using the EYFSP total score, but that further work on the EYFSP still needs to be done for it to be considered psychometrically valid in other ways. Replacing the EYFSP with another measure would bring a significant challenge and overhaul to the educational system and require a significant programme of work to either revise the EYFSP or replace it with something similar. We would suggest avoiding this until the measurement properties of the EYFSP have been further explored. We have now explained this in our discussion, which we feel makes our implications clearer p.36).
