# Supplementary material for: An assessment of the teacher completed ‘Early Years Foundation Stage Profile’ as a routine measure of child developmental health
Source: PLoS One. 2025 Mar 19;20(3):e0302771. doi: 10.1371/journal.pone.0302771 (PMC11957556; doi:10.1371/journal.pone.0302771)
Supplement: Attachment — Submitted filename: Early Years Foundation Stage Profile.docx [file pone.0302771.s004.docx]

Early Years Foundation Stage Profile (EYESP)

Interesting article that I enjoyed reviewing ---thanks.

Please look up the manuscript guidelines for Plos One

You need to clean up spacing and when you indent and when you do not indent the first paragraph after a heading

Only three levels of headings are permitted

The references need to be reformatted.

Your discussion is really results that should be more clearly related to your hypothesis

Then in the discussion it should bring in the lit review with your findings

**Small issues**

In the marked up version ( there are no track changes) page 25 I found this (45,48). (47)

Not sure why the 47 is outside the period

A change in 6 in the EYFSP total score results in a change of -1.22 in SDQ, and a change in 6 in EYFSP-

**Seems it should be of 6 in the EYESP (in both places)**

**On page 44**

**Despite this, this study**

**----avoid the double this page 50**
